# Extracellular Vesicles Could Carry an Evolutionary Footprint in Interkingdom Communication

**DOI:** 10.3389/fcimb.2020.00076

**Published:** 2020-03-03

**Authors:** Ricardo Correa, Zuleima Caballero, Luis F. De León, Carmenza Spadafora

**Affiliations:** ^1^Center of Cellular and Molecular Biology of Diseases, Instituto de Investigaciones Cientificas y Servicios de Alta Tecnologia (INDICASAT AIP), Panama, Panama; ^2^Department of Biotechnology, Acharya Nagarjuna University, Guntur, India; ^3^Department of Biology, University of Massachusetts, Boston, MA, United States

**Keywords:** extracellular vesicles, *Plasmodium falciparum*, interkingdom, communication, evolution, parasites, host, vector

## Abstract

Extracellular vesicles (EVs) are minute particles secreted by the cells of living organisms. Although the functional role of EVs is not yet clear, recent work has highlighted their role in intercellular communication. Here, we expand on this view by suggesting that EVs can also mediate communication among interacting organisms such as hosts, pathogens and vectors. This inter-kingdom communication via EVs is likely to have important evolutionary consequences ranging from adaptation of parasites to specialized niches in the host, to host resistance and evolution and maintenance of parasite virulence and transmissibility. A potential system to explore these consequences is the interaction among the human host, the mosquito vector and *Plasmodium* parasite involved in the malaria disease. Indeed, recent studies have found that EVs derived from *Plasmodium* infected red blood cells in humans are likely mediating the parasite's transition from the asexual to sexual stage, which might facilitate transmission to the mosquito vector. However, more work is needed to establish the adaptive consequences of this EV signaling among different taxa. We suggest that an integrative molecular approach, including a comparative phylogenetic analysis of the molecules (e.g., proteins and nucleic acids) derived from the EVs of interacting organisms (and their closely-related species) in the malaria system will prove useful for understanding interkingdom communication. Such analyses will also shed light on the evolution and persistence of host, parasite and vector interactions, with implications for the control of vector borne infectious diseases.

## Introduction

The evolution of cell communication is crucial in determining how organisms respond to and interact with each other and their environment. Protozoan parasites also have the ability of releasing membrane–enclosed vesicles toward their extracellular space. These small bodies are collectively known as “extracellular vesicles” (EVs) and can vary in size, composition and origin (Lener et al., 2015). EVs can function as signal carriers for single cell-cell communication or even more complex interactions at the level of tissues and organs (Kim et al., 2015). In this review, we present an overview of what is currently known about these extracellular bodies, the findings reported on protozoarian communication through EVs, their role in pathogenesis, program cell death, communication, and interkingdom coevolution.

## Historical Perspective

Despite the use of EV terminology for more than 30 years, starting with the description of exosomes from reticulocytes (Harding et al., 1984), one of the first reports of what would be later referred to as EVs came from the activity of “thromboplastic proteins of the blood” (1946) (Chargaff and West, 1946). These proteins were composed of nanometric vesicles, and were later denominated “platelet dust” (1967) (Wolf, 1967). Other studies were also performed to investigate the activity of cartilage matrix vesicles in bone mineralization in 1969 (Anderson, 1969). Not long after, in 1971, small vesicles originating from RBCs infected with *P. falciparum* were observed using electron microscopy (Luse and Miller, 1971). Later on, studies on the regulatory capacity of EVs from T cells on the immune response triggered a renewed interest due to the potential therapeutic uses of EVs (Raposo et al., 1996). Important advances in the understanding of the role of EVs began to emerge with the identification of key functions such as their role in horizontal genetic transfer, modulation of the immune response, and cell differentiation. This multifunctional activity of EVs stems from their capacity to transport a wide range of distinct biomolecules that can alter the biological functions of target cells (Kalra et al., 2016). Currently, the application of high throughput sequencing tools enables the acquisition of massive data on EV populations from a diverse source of cellular and tissue models, offering new opportunities for the development of novel applications.

EVs have been grouped in a variety of ways (van der Pol et al., 2012; Akers et al., 2013; Raposo and Stoorvogel, 2013; Yanez-Mo et al., 2015; Szatanek et al., 2017). Briefly, EVs originate from cell sacs that are essentially comprised of thousands of different proteins and unique lipids, and which contain not only DNA and mRNA but also small nucleolar RNA (snRNA), Y RNA, mitochondrial RNA, vault RNA and long ncRNA (non-coding RNA) (Lazaro-Ibanez et al., 2014; Kreimer et al., 2015; van Balkom et al., 2015). Thus, EVs have been classified into exosomes, microvesicles and apoptotic bodies, depending on their origin, size and molecular composition. For instance, exosomes range in size from 50 to 150 nm. They are produced by invagination of the endosomal membrane during maturation of multivesicular bodies, and are released outside the cell after fusion with the plasma membrane (Keller et al., 2006; van Niel et al., 2018). Exosome formation is associated with specific proteins located in the endosome, such as tetraspanins, chaperones, and the Rab GTPase family (Ostrowski et al., 2010). A primary component of exosomes is the endosomal sorting complex required for transport (ESCRT), which is involved in the formation of exosomes in the late endosome and in the transportation of cargo (Raposo and Stoorvogel, 2013).

On the other hand, micro vesicles (MVs) are produced after budding directly from the plasma membrane. They have often been referred to in the literature with different names, such as ectosomes, microparticles, or shedding vesicles (Meldolesi, 2018). MVs are formed in cytosolic microdomains produced by the redistribution of phospholipids of the interior side of the plasma membrane, and then released to the extracellular space after vesicle fission (Cocucci and Meldolesi, 2015). MVs have sizes that range from 0.1 to 1 μm, which overlaps with the reported size of exosomes. This indicates that size is not a reliable criterion to differentiate between EVs. In living cells, the redistribution of lipids is facilitated by translocases that allow the movement of phospholipids in both directions across the plasma membrane, such as phophatidyl serine, which induces membrane budding and generation of MVs (Leventis and Grinstein, 2010; van der Heyde et al., 2011; Mantel and Marti, 2014). Additionally, other changes in the endosome and the plasma membrane are involved in the production of MVs, such as overexpression of GTP-binding ARF factor 6 (ADP-ribosylation factor 6), the formation of the complex VPS ATPse E3 ligase, and the interaction of the tumor susceptibility gene 101 (TSG101) with arrestin domain-containing protein 1 (ARRDC1). These modifications produce contractions in the cytoskeletal arrangement and the interaction with phospholipases result in the release of MVs (Muralidharan-Chari et al., 2009; Nabhan et al., 2012).

Finally, apoptotic bodies are released only when apoptosis is triggered in a healthy cell, beginning with chromatin condensation and blebbing of the membrane, followed by proteomic degradation and releasing of apoptotic bodies to the extracellular space (Elmore, 2007). Apoptotic bodies have a larger size in comparison with other types of EVs, ranging from 50 to 5000 μm (Nawaz et al., 2014). Smaller vesicles can be embedded within apoptotic bodies, and may enclose organelles as well as fragmented nuclei, a feature that uniquely separates these particles from other types of EVs. It is still unknown how these smaller vesicles are formed (Bergsmedh et al., 2001; Akers et al., 2013), but the blebbing mechanism of apoptotic bodies has been associated with actin-myosin interaction (Coleman et al., 2001).

## EV Characteristics During Pathogen Infection

Intracellular and free-living pathogen-derived EVs carry signals that may mediate pathogen-pathogen communication. These signals modulate host biological functions, and induce the production of EVs by host effector cells that have been stimulated by direct contact with the pathogens or by contact with their EVs (Mantel and Marti, 2014). Special attention has been paid to the EVs of intracellular pathogens because this type of pathogen can modify the molecular composition of EVs, changing their function upon release from an infected host cell. For example, macrophages are induced to release inflammatory cytokines after taking up exosomes that were released from other macrophages infected with *Mycobacterium avium* (Bhatnagar and Schorey, 2007). EVs from a wide variety of other pathogens have important activity during infections, such as those produced by *Giardia, Chlamydia, Trichomonas*, and *Cryptococcus* (Benchimol, 2004; Rodrigues et al., 2008; Frohlich et al., 2012; Twu et al., 2013). Similarly, intracellular protozoan parasites also have an impact on the host, by altering its immune response. For instance, EVs derived from *Toxoplasma gondii* are known to induce murine macrophages to release inflammatory proteins such as TNF-α, iNOS, and IL-10 (Silva et al., 2018).

## EVs in Trypanosomatids

In infections caused by protozoan parasites, the release of EVs can be observed throughout most of the parasite's life cycle, suggesting that EVs are a fundamental component of parasitic infection. As previously reported, EVs are involved in the host–parasite interaction and in communication between parasites, inducing dysfunction in the immune responses or manipulating the physiology and metabolism of the host (Silvester et al., 2017). EVs have been identified in some of the most pathogenic protozoans, those responsible for some of the most widespread, lethal and disabling vector–borne diseases, such as Chagas disease, African trypanosomiasis, and leishmaniasis. Their causative parasites have a complex life cycle, undergoing several transformations during their asexual and sexual stages. As explained below, new findings have determined that EVs play a role in a variety of infective processes, including the immune response of the hosts. The principal findings on the parasite-derived EVs from these major vector–borne diseases are summarized below.

*Trypanosoma cruzi*, the etiological agent of Chagas disease, infects macrophages during its trypomastigote stage and can generate both exosomes and ectosomes during infection (de Pablos Torro et al., 2018). The parasite then invades new cells and tissues after cellular differentiation and replication. It has been shown that EVs are present in almost all stages of infection (Bayer-Santos et al., 2013). In addition, the modulating activity of EVs is determined by the phase of the disease (acute or chronic), the immune status of patients and the parasite load during infection. During *T. cruzi* infection, EVs can be released from parasites or host cells and can be taken up by both parasites and host cells. For instance, Ramirez et al. (2017) showed that the integration rate of EVs with THP1 monocyte cells is significantly higher upon release from human tissue–cultured trypomastigotes than from insect stage epimastigotes or metacyclic trypomastigotes. If these infected monocyte-derived EVs fuse with other trypomastigotes, they can offer them protection against lysis from the complement system action (Cestari et al., 2012; Ramirez et al., 2017). Likely, this protection originates after trypomastigotes and immune complexes interact with each other through their EVs (Diaz Lozano et al., 2017). The increased incorporation of EVs through a Ca^2+^-dependent mechanism, in comparison with other stages, is caused by a higher rate of redistribution of phosphatidylserine (PS) in THP-1 monocytes upon interaction with tissue-culture trypomastigotes (Ramirez et al., 2017). Therefore, similar to other cell models, the modulation of the immune response using EV-cargo signals relies on the key role played by PS in facilitating the uptake of EVs from infected host cells. Nevertheless, more studies are required to describe the precise signal cascades involved in the Ca^2+^ efflux and the release of EVs during *T. cruzi* infection. Interestingly, *T. cruzi* proteins are found inside host cells after their incorporation of *T. cruzi* EVs, and this insertion appears to be targeted at specific cells types such as fibroblasts, muscle and neuronal cells. In fact, parasite proteins have not been detected in other types of cells such as lymphocytes or erythrocytes (de Pablos Torro et al., 2018). EVs fusion with THP1 cells produce a higher expression of the genes IKBKB, NR3C1, and TIRAP, which have been associated with an increased generation of reactive oxygen species (ROS) and nitric oxide (NO) (Chowdhury et al., 2017). Additional evidence of the role of EVs in the pathogenicity of trypanosomatids was offered by Trocoli Torrecilhas et al. (2009) who demonstrated that the parasitaemia in BALB/C mice increased following the inoculation of *T. cruzi* EVs prior to infection. Notably, proteomic analysis of EVs from trypomastigotes (host stage) and epimastigotes (vector stage) of *T. cruzi* revealed that most (70%) of the identified EV proteins are present in both stages.

In a similar scenario, EVs play an important role during infection by *Leishmania* parasites, which are transmitted to humans by sandfly bites. Most of the research on EVs from *Leishmania* species has been performed using *L. major* and *L. donovani*, which have different clinical manifestations, with the former developing in the skin and the latter, viscerally. Following the transformation of promastigotes to amastigotes inside macrophages in *in vitro* studies, it has been shown that EVs may be released to the supernatant and present virulence factors such as zinc-metalloproteinase gp63 (Silverman et al., 2008). This protein activates the tyrosine phosphatase SHP-1, which has an inhibitory effect on the pro-inflammatory cytokine response activity of macrophages after inhibiting the host IFN-γ/Jak-STAT1 cascade and the p38-MAPK signaling pathway (Gomez et al., 2009; Mantel and Marti, 2014).

In hepatic cells, protein gp63 also inhibits the enzyme Dicer1, causing the downregulation of the miRNA miR-122, which provokes a change in the lipid metabolism of the host, contributing to the survival of the parasite (Descoteaux et al., 2013). In addition, gp63 participates in the activation of signaling proteins and transcription factors that might be involved in the up/down regulation of several genes in the target macrophage (Hassani and Olivier, 2013). It is not clear yet, however, whether this gp63 action is mediated by EV integration into hepatic cells (Ghosh et al., 2011). On the other hand, EVs from promastigotes have been shown to modify the immune response by decreasing the production of IL-8 and TNF-α, increasing IL-10 and eliminating the dendritic cell capacity to activate Th1 cells (Silverman et al., 2010). In a recent report, EVs were associated with the increased production of cytokines in macrophages and B1 cells as well as with several virulence factors in *L. amazonensis* (Barbosa et al., 2018). What is clear in infections by *Leishmania* parasites is that the EVs from both intracellular and free-living parasites are modulating the immune response of the human host.

## EVs in Malaria Pathogenesis

An early report on malaria EVs focused on their capacity to induce thrombocytopenia, which is associated with cerebral malaria (CM) (Piguet et al., 2002). Later, it was shown that *P. falciparum* EVs were also present in the sera of infected patients, establishing the occurrence of increased level of EVs in patients ridden with this species of *Plasmodium* (especially associated with coma cases in CM) in comparison with *P. malariae* and *P. ovale* infections (Combes et al., 2004; Pankoui Mfonkeu et al., 2010). In the case of *P. vivax* infections, patients also show elevated levels of EVs derived from erythrocytes, platelets, leucocytes and monocytes, relative to healthy individuals (Campos et al., 2010). Proteomic analyses of circulating EVs in infected patients have identified proteins related to parasite metabolism, invasion and pathogenesis, such as enolase, Hsp 90, lactate dehydrogenase, ADP-ribosylation factor 1, phosphoglycerate kinase and merozoite surface protein 1 (Antwi-Baffour et al., 2016).

Animal models have also served to study the relationship between EVs and malaria pathogenesis and to gain insights into the corresponding host immune response. For example, the high levels of iRBC (infected red blood cell)-derived EVs during CM were reduced after inhibition of the ATP-binding cassette transporter ABCA1 gene, which is a known mediator of MV formation. Specifically, this ABCA1 inhibition reduced TNF-αlevels in sera and cell sequestration of leukocytes into the brain, both features of complicated malaria (Combes et al., 2005). Remarkably, it has been shown that the particular haplotypes of the ABCA1 promoter determine the levels of EV secretion during infections, thus increasing or decreasing the virulence of the parasite (Sahu et al., 2013). Mice models have been used to study the immune response, particularly the inflammatory processes related to macrophage activation in a TLR–dependent manner after *P. berghei-*derived EVs were inoculated into the animals (Couper et al., 2010). Recently, Shrivastava et al. (2017) reported that RBC-derived EVs could be internalized massively by astrocytes along with high levels of CXCL10, a known marker of CM, in *P. berghei*–infected mice (Shrivastava et al., 2017).

On the other hand, after hand *in vitro* studies of *P. falciparum* have also informed the biological function of EVs. For instance, in a model of CM, platelet–derived EVs were associated with the transference of platelet antigens into iRBCs using a PfEMP1-dependent pathway, which resulted in an increase in the binding of infected cells with the endothelium (Faille et al., 2009). In 2013, two independent studies found that *P. falciparum* uses EVs for parasite-parasite and host-parasite communication (Mantel et al., 2013; Regev-Rudzki et al., 2013). These studies elegantly demonstrated several PfEV activities, such as activation of neutrophils, secretion of both pro- and anti-inflammatory cytokines following EV uptake by macrophages, induction of gametocytogenesis, and incorporation of DNA plasmids, which can spread drug resistance among naïve parasites. In addition, MV markers stomatin, GTP-binding protein ARF6 and the disassembly factor VPS4 were identified as cargo of the EVs using proteomic analysis (Mantel et al., 2013). In the context of these findings, the evidence suggested that EVs could be used as indicators during *P. falciparum* infections to estimate parasite density, serve as signals in parasite–parasite communication, and regulate the period of transmissibility to vectors (Mantel and Marti, 2014). Interestingly, recent proteomic analysis identified several proteins that may participate in the invasion of RBCs using molecular mimicry of the host molecules. Particularly, the ring–exported protein 2 (Rex2) and the *Plasmodium-*exported element (PEXEL) present a high molecular similarity to the human RAC2 protein family (Barteneva et al., 2013). Recently, it was also shown that human NK cells activated by iRBC–derived EVs respond by exposing exposed MDA5, a pathogen recognition receptor (Ye et al., 2018).

The origin of *P. falciparum* EVs has been described in asexual stages. Maurer's clefts are important erythrocytic structures formed after invasion by the parasite, participating in protein trafficking and vesicle formation directed at the erythrocyte plasma membrane (Spycher et al., 2006). This was demonstrated when Maurer's cleft proteins were reported as components of released EVs during *P. falciparum in vitro* culture (Mantel et al., 2013). The molecular deletion of Maurer's cleft proteins inhibited the production of EVs, confirming the role of this host-parasite structure in vesicle biogenesis (Regev-Rudzki et al., 2013). Further findings confirmed the participation of the EV cargo in host immune modulation, host vascular function manipulation, gametocytogenesis and gene regulatory functions through small RNAs, DNA, and specific interacting components associated with severe malaria (Mantel et al., 2016; Sisquella et al., 2017; Babatunde et al., 2018). Altogether, these findings revealed that malaria parasites use EVs as an effective communication system that act like cellular “carrier pigeons” to regulate multiple functions of both the parasite population and host homeostasis.

## Individual Suicide or Collective Homeostasis? the Role Of EVs

Traditionally, programed cell death (PCD) is defined as a genetically regulated mechanism that is used by multicellular organisms for homeostasis and development (Gunjan et al., 2016). However, in recent years, there has been increasing interest in death regulation in protozoan parasites, generating debate around the traditional classification of programed cell death (PCD) pathways (Proto et al., 2013). In fact, new evidence shows that intracellular parasites can suffer apoptosis (Li et al., 2015; Mandal et al., 2016; de Castro et al., 2017; Gunjan et al., 2018), indicating that PCD is not unique to multicellular organisms. Thus, understanding of the PCD mechanisms that take place during microbial infections is an exciting field of study, given that death signals might be used in therapeutic treatments. For example, *Plasmodium, Trypanosoma*, and *Leishmania* parasites present PCD features under distinct experimental conditions (Duszenko et al., 2006; Gunjan et al., 2016; Mandal et al., 2016; Basmaciyan et al., 2017; de Castro et al., 2017; Wei et al., 2018).

Similar to multicellular organisms, these parasites present classic PCD markers, such as DNA fragmentation, cell shrinkage, chromatin condensation, PS translocation and loss of the mitochondrial membrane potential (Reece et al., 2011). Furthermore, several effectors have been suggested to induce apoptosis in protozoan parasites. For example, prostaglandin D_2_ is involved in PCD in *Trypanosoma brucei* procyclic forms, by generating ROS (Duszenko et al., 2006). The description of the gene metacaspases 1 (PfMCA1) and the use of pan caspase inhibitor z-VAD-fmk confirmed the presence of PCD in *P. falciparum* and in *P. vivax* (Meslin et al., 2007; Rezanezhad et al., 2011). In addition, it has been shown that PfMCA1 is regulated by the C2 domain of the calcium-dependent membrane targeting domain and a CARD domain (Caspase Recruitment Domain) (Sow et al., 2015). In *Leishmania* the role of MCA has been identified as being a precursor of PCD, acting on the autophagic protein ATG8 (Casanova et al., 2015). The above evidence makes it clear that protozoan parasites do indeed have PCD mechanisms, albeit distinct from those of multicellular organisms.

Remarkably, EVs can also induce apoptosis during stress and normal development conditions by carrying Fas and TRAIL to different target cells (Janiszewski et al., 2004; Stenqvist et al., 2013). Recently, our group reported that parasite-derived EVs carrying lactate dehydrogenase can induce PCD in *in vitro* cultures of *P. falciparum* during high parasitaemia levels, showing that parasites can likely sense each other and release EVs to regulate the parasite population (Correa et al., 2019). If apoptosis induction involves the pathogen communicating with its kind through EVs, as the evidence suggests, this would represent an important evolutionary force in the adaptation of parasitism to specialized niches in the host. EVs could be involved in the communication strategies used by the parasite to manipulate its hosts and vector in order to maintain its virulence, survival and transmissibility. There is increasing evidence from various fields and approaches suggesting that the study of EVs might help understand the complexity of mammalian host-parasite-vector interactions in protozoan pathogens. We suggest that an integral molecular analysis of EVs could provide a genetic marker(s) that would help answer certain mysteries present in the study of the evolution of interkingdom communication and its role in the adaptation of parasite species and that *P. falciparum* could be a good model to test this hypothesis.

In the case of the malaria parasite, it has been suggested that a mechanism of self-regulation may exist to limit the intensity of the parasitaemia in *in vitro* cultures. Interestingly, in those conditions, the parasitaemia growth of *P. falciparum* can reach a maximum multiplying factor of 8, which is far below a potential factor of 16 (Deponte and Becker, 2004) under ideal conditions. Based on this behavior, it had been suggested that *P. falciparum* uses mechanisms of self-regulation in response to density stress (Mutai and Waitumbi, 2010), and these mechanisms might depend on EVs mediated apoptosis. For instance, recent studies have observed programmed cell death in highly parasitized *in vitro* cultures of *Plasmodium*, and another study that has identified molecules involved in the signaling of death (Totino et al., 2014; Engelbrecht and Coetzer, 2016; Chou et al., 2017; Correa et al., 2019).

## The Possible Role of EVs in Evolution by Horizontal Gene Transfer

**Figure 1:**
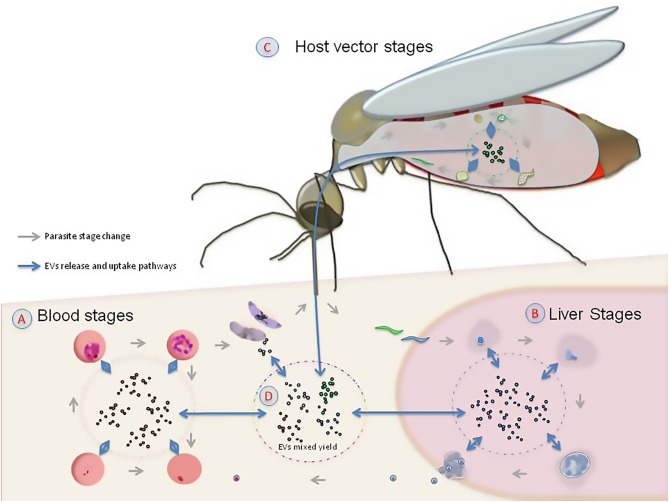
The role of extracellular vesicles (EVs) in the life cycle of *Plasmodium falciparum*. Given that EVs are produced by the parasite and the hosts in the human erythrocyte **(A)**, the liver **(B)**, and the mosquito vector **(C)**, they are likely to play a signaling role in the interaction among host, parasite and vector **(D)**.

The basic biochemical composition of EVs includes a phospholipid membrane that forms a sac and can contain conserved proteins, such as enzymes, growth factors, different types of receptors and cytokines, as well as DNA, coding and non-coding RNA and several metabolites (Raposo and Stoorvogel, 2013). Since their discovery, EVs derived from red blood cells infected with *P. falciparum* generated interest regarding their potential role in cell signaling, intra-regulation and communication between host and pathogens (Mutai and Waitumbi, 2010; Proto et al., 2013). Recently, Sundararaman et al. (2016) suggested that EVs could play a role in DNA recombination in the asexual stage of the infection inside the erythrocytes. This indicates that the study of EVs in *P*. *falciparum* could provide an opportunity to understand host-parasite and parasite-parasite interactions. In particular, its ability to grow in laboratory cultures and its specialized life cycle confined to mosquitoes and humans, makes *P. falciparum* an optimal model system for the study of the evolution of inter-kingdom communication (Figure 1).

A series of molecules derived from EVs have been involved in cell signaling and intra-regulation (Mutai and Waitumbi, 2010; Proto et al., 2013), including a variety of proteins and genetic material, and, potentially, transposons (Lefebvre et al., 2016; Preusser et al., 2018), all of which are known to mediate communication between host and pathogens (Schorey et al., 2015). For instance, recent studies on *P. falciparum* show that EVs carry proteins and nucleic acids potentially involved in the horizontal transfer of information parasite↔parasite and/or parasite→host (Mantel et al., 2013, 2016; Regev-Rudzki et al., 2013). In addition, the release of EVs from iRBCs has been involved in signaling the transition of the parasite from the asexual to sexual stage as well as its sequestration in micro-capillaries, both of which are transitions that would favor vector transmission (Aingaran et al., 2012; Cornet et al., 2014).

Interkingdom communication via EVs could also play a role in coevolution of interacting organisms. However, the coevolution of cell signaling between different species, such as host-pathogen interactions or mixed infection by related pathogens has not been considered so far. Mixed infections of multiple parasites within a host are common in nature (Krishna et al., 2015; Bernotiene et al., 2016; Graystock et al., 2016). For instance, great apes, and even humans, can host several species of *Plasmodia* parasites (Prugnolle et al., 2010). EVs could mediate communication by facilitating horizontal transfer of genetic information between different *Plasmodia* species (Regev-Rudzki et al., 2013; Kawamura et al., 2017; Otto et al., 2018; Ben-Hur et al., 2019; Plenderleith et al., 2019). Several lines of evidence support this possibility. Indeed, the frequency of EVs found in pathogens suggests that EVs are common in interactions between host and parasites, and are likely to modify the biological functions of the host (Mantel et al., 2016; Zhu et al., 2016). In addition, molecules derived from EVs associated with host-pathogen interactions are likely to show a footprint of the reciprocal selection between host and pathogens (Kawamura et al., 2017).

## Evolutionary Consequences of Interkingdom Communication

The evolution of interkingdom communication could have several consequences. For example, information transfer in a host with mixed infections (Mantel and Marti, 2014) could facilitate the stable co-existence of multiple pathogen species. This is likely the case in mixed infections of *Plasmodia* spp. in apes, which could be less virulent than a solo *P. falciparum* infection in modern humans, as reported in van Niel et al. (2018). However, it is not yet clear how universal mild infection in apes hosting mixed infections of *Plasmodia* spp. is. Similar to host and parasites, EVs could also facilitate signaling between the *Plasmodium* parasite and the mosquito vector (Deolindo et al., 2013; Twu et al., 2013; Bayer-Santos et al., 2014; Montaner et al., 2014). This information exchange is likely to have played a key role in the initial approach between host and parasite that ended up in the establishment of the infection (Figure 1D). However, an integrative examination of EVs derived from infected host, parasite and vector is necessary to shed light on the origins of *P. falciparum* and the development of malaria pathogenesis.

## Testing for the Role of EVs

To test for the role of EVs in the evolution of host-parasite interaction, a deeper analysis of the properties and molecular composition of EVs is necessary. Proteins derived from EVs have already been suggested to mediate communication between host and pathogens, especially in intracellular microorganisms such as *Trypanosoma* spp. (Matthews, 2005; Garcia-Silva et al., 2014), *Leishmania* spp. (Silverman and Reiner, 2011) and *Plasmodium* spp. (Martin-Jaular et al., 2011; Regev-Rudzki et al., 2013). In *Plasmodium* spp., an important number of the molecules have recently been identified (Batista et al., 2011; Correa et al., 2017). These molecules include a wide range of proteins that are involved in metabolism and immune processes. Sequence variation in these EV proteins and their interacting counterpart in hosts could be examined across tissues infected by the parasite, such as host erythrocytes and mosquito gut lining and salivary glands. Sequence data from these proteins, as well as nucleic acids (e.g., DNA, RNAs) derived from EVs, can be used to infer parallel changes associated with coevolution between parasites and hosts. Some ideas are given in Table 1. Additional analyses of these EV molecules could explore patterns of selection acting on aminoacid (or nucleotide) sequence in hosts and parasite. These analyses could be performed using cutting-edge sequencing tools in combination with readily available comparative phylogenetic methods such as parsimony, maximum likelihood, and Bayesian inference (Yang and Bielawski, 2000). From a phylogenetic perspective, comparative analyses could include EV molecules derived from host, parasite and vectors, as well as their closely-related species. These analyses could also reveal whether the communication mechanism via EVs represents a conserved strategy across plasmodia-insect-mammalian species. Finding a conserved communication strategy via EVs could explain the unusual ability of New World mosquitoes to transmit the *Plasmodium* parasite, even though American mosquitoes diverged from their African counterparts 95 million years ago (Molina-Cruz and Barillas-Mury, 2014).

**Table 1:** Could EVs mediate key aspects of coevolution between parasite and hosts?

**Potential role of EV_***S***_ in host-parasite interaction**	**Experimental approach**	**Prediction**
Information (i.e., molecules) delivered by parasites via EVs plays a role in communication that is specific to its host, parasite and vector system.	Characterization of “EVsome” by differential proteomic analysis (e.g., nano LC Mass Spectrometry) and molecular analysis (e.g., DNA, RNA) to identify key signaling molecules from distinct parasite stages of its life cycle.	The composition of EVs has been previously described as “hybrid” (Mantel et al., 2013; Correa et al., 2019) due to the presence of host and parasite proteins. We expect that the composition of EVs can be distinctively associated with its source system (e.g., host, parasite, or vector), suggesting a potential role in information transfer.
EV cargo has functional consequences for the interaction between hosts and parasites.	Experimental assays such as coprecipitation can be used for screening and identification of interacting EV proteins between host and parasite. This will help explore the functional role of interacting EVs components in host and parasite. *In vitro* analyses of gene expression could help identify mRNA molecules in parasite EVs with a role in specific signaling pathways.	Parasite proteins secreted by EVs interact with receptors of the host cell. This interaction leads to detectable changes in enzyme activity, metabolite production, phenotypic change, gene expression, or signaling pathways. Genetic material in the cargo of EVs influence the function of host cells.
The signaling role of EVs could affect coevolution between hosts and parasites.	(i) A comparative analysis of macromolecules in the cargo of EVs of different *Plasmodium* spp. could highlight specific variations as a consequence of an adaptation to the infection of different mammalian hosts. (ii) Homologous sequences of the EVs molecules (e.g., protein, DNA, RNA) across species of the parasite (*Plasmodium* genus) as well as EVs receptors of the host species can be studied using phylogenetic comparative analysis.	Phylogenetic analyses may show coupling of the evolutionary history between parasite proteins secreted by EVs and the receptors of the host cell.

The analyses suggested in Table 1 might exploit whole-genome sequence tools that have been used recently to study the coevolution of host-parasite interactions. In the absence of known rare genomic changes or other idiosyncratic markers, whole-genome analyses provide a useful tool to circumvent studies with single or low number of markers (Froeschke and von der Heyden, 2014). One example is the use of linkage mapping to perform genetic crosses, useful for localizing phenotypic traits in a range of organisms. Linkage mapping has been applied to *P. falciparum* to identify key genes related to drug resistance and cell development (Ranford-Cartwright and Mwangi, 2012). This method has made it possible to map genes, causing a given phenotype, to genome regions and to find the responsible genes and/or mutations by performing functional genomics experiments (Anderson et al., 2018). Additionally, there are a number of next-generation sequencing tools that can be used to explore the components of the EV cargo as well as the evolutionary history of host-parasite interaction mediated by EVs. This includes parallel sequencing of known (and novel) peptides and nucleic acids derived from the EVs host and parasite. These analyses can also be targeted to specific cell types (e.g., via single cell parallel sequencing), which could facilitate the functional characterization of host and parasite EVs *in vitro* (Turchinovich et al., 2019).

To date, most studies have focused on the interaction between mammalian host and parasites. Nevertheless, the role of parasite EVs in vector attraction to infected cells has received less attention (Figure 1). The study of these interactions could help explain the observation that infected mosquitoes can be manipulated by *Plasmodium* in a way that enhances vector attraction to human odor (Lacroix et al., 2005; Smallegange et al., 2013; Batista et al., 2014). In this interaction, a parasite-driven regulation of vector genes (Lu et al., 2007) might activate the receptors used by mosquitoes to detect hosts (Takken and Knols, 1999). EVs could mediate the attraction to the infected hosts by influencing both gene regulation and activation of other signals during infection (reviewed in Barteneva et al., 2013; Mantel and Marti, 2014). While understanding these signaling pathways is challenging because of the inherent difficulties of studying intracellular pathogens, the specialized nature of the *P. falciparum* life cycle presents a unique opportunity to explore this issue.

A number of reports that suggest coevolution between microbes and hosts can be found in literature (Chapman and Hill, 2012; Mozzi et al., 2018; Quintana-Murci, 2019). The importance of polymicrobial interactions in this occurrence has also been presented (Rioux et al., 2007). Thus, the possible role of EVs on these events would only add another piece to explain coevolution.

## Conclusions

The functional role of EVs has been associated with intercellular communication. However, recent evidence indicates that EVs are also ubiquitous in the interaction among mammalian hosts, parasites and vectors. This suggests that EVs play an important role in interkingdom communication. Thus, communication through EVs is likely to have important evolutionary consequences such as the adaptation of parasitism to specialized niches in the host. EVs could also be involved in the communication strategies used by the parasite to take advantage of its mammalian host and its vector to maintain its virulence, survival and transmissibility. We suggest that the *Plasmodium* parasite involved in the malaria disease represents a potential model system to explore the role of EVs in the evolution of host-parasite interaction and interkingdom communication. Deciphering the cargo composition of EVs derived from parasites, hosts, and vectors will also inform the evolution of the pathogenicity of malaria and other protozoan parasites.

## Author Contributions

RC conceived the idea and drafted the initial manuscript. ZC and LD wrote sections of the manuscript. RC, ZC, and CS reviewed and worked to produce the final concepts. CS put together the final draft. All authors read, revised, and approved the final manuscript before submission.

### Conflict of Interest

The authors declare that the research was conducted in the absence of any commercial or financial relationships that could be construed as a potential conflict of interest.
